# Supplementary material for: Endogenous Ethanol and Triglyceride Production by Gut Pichia kudriavzevii, Candida albicans and Candida glabrata Yeasts in Non-Alcoholic Steatohepatitis
Source: Cells. 2022 Oct 27;11(21):3390. doi: 10.3390/cells11213390 (PMC9654979; doi:10.3390/cells11213390)
Supplement: Supplementary file 1 [file cells-11-03390-s001.zip › Supplementary data R2.pdf]

Endogenous Ethanol and Triglycerides Production by Gut *Candida* and *Pichia* Yeasts in Non Alcoholic Steatohepatitis

SUPPLEMENTARY DATA

Review of the literature for auto-brewery syndrome (ABS)

In this work, we sought to better describe the auto-brewery syndrome that has been recently linked with nonalcoholic steatohepatitis (NASH) [1]. For this, we used several keyword as 'auto-brewery syndrome', 'endogenous ethanol production', 'endogenous ethanol fermentation', 'gut fermentation syndrome', 'drunkenness disease', 'ethanol' or 'Meiteisho' as first reported by Sato in 1952 [2]. Our research was not restricted by year of publication. We used the Pubmed, Google scholar and Google database.

In total, we had found 59 cases (all references mentioned in Supplementary Table S1) since 1952. Most of cases were reported in Japan (n = 40, 68%) and USA (13, 22%). The other countries of origin were China (2 case), Belgium, Italy, Sweden and Turkey (1 case each). Median age was 41 years (interquartile range [26-49], range 1 – 75 - Mean 38.86 ± 18.65 years). Male were overrepresented (Male 38 (64%), female 20 (34%)) – for one case, gender was not determined.

Interestingly, 2 patients presented ABS after alcoholism withdrawal [3,4], history of fatty liver was reported in 1 case [5] 1 had cirrhosis [6]. Specifically in USA, ABS was reported several times in patients with metabolic disease such as obesity (5 case, 45-65 years, all from USA) and diabetes (4 cases, 45-61 years, all from USA). However, this syndrome has been reported both in normal-weight and overweight/obese patients (body mass index ranging from 16 to 45 kg/m2).

Apart from patients with metabolic disease mainly reported from USA, we found several cases (n = 21) with gut medical or surgical history (Crohn's disease, short bowel syndrome, gut surgery) associated with children and Japan.

In 6 cases a triggering event was found. In all these 6 cases, antibiotics treatments (amoxicillin-clavulanic acid n = 2, cephalexin n = 1, antibiotics not known for other cases) was the triggering event.

Complications of ABS can be very severe. Indeed, we identified 2 traffic accidents, 2 seizures, 1 confusion, 1 coma, 1 fall complicated with intracranial bleeding, 1 pancreatitis, and 1 massive bowel dilation.

Liver plasmatic markers were available for 4 patients. In 3 of them, they were elevated and in 1 case the liver plasmatic marker was normal but liver echography deciphered liver steatosis. For 4 patients, liver echography was available and in all 4 cases evidencing liver steatosis (all male, 30-47 years, not from Japan) [3,7,8,9]. Liver biopsy was performed in 2 cases, both from China showing steatohepatitis in the 2 cases [1,8], associated with liver fibrosis in 1 case [1].

We have also found blood ethanol measurements for 12 cases. The maximal ethanol blood level for each reported cases ranged from 0.64 – 4 g/L. Accordingly, all of them had a maximal ethanol blood level > 0.5 g/L, which is the upper limit for car driving in France. Most of cases harbored very high ethanol levels between 2 and 4 g/L (median 3.16 [2.39 – 3.97] range 0.64 – 4.1 g/L).

We review also the microbiological results of the 59 cases. For 3 patients, no microbiological data was available. Among these 3 patients without microbiological investigation, 1 patient was cured with antifungal treatment, thus suggesting a fungal etiology. For 56 patients, microbiological data were provided. In only 1 case, no fungus nor identified pathogenic bacterium was found in stool. For the other 55 cases, at least one yeast and/or specific bacteria was found.

The very recent report by Yuan *et al*. [1] identified only *K. pneumoniae* without any yeast (specific culture and ITS) and the patient did not respond to antifungal treatment confirming the non-fungal origin of the disease in this specific case. We were struck that in this seminal study on endogenous ethanol production, ABS and NASH, yeasts were not tested for all samples and patients. Apart this exceptional case, we found 54 cases with at least one yeast. In most cases, the yeast was isolated from the gut (upper or lower, 53/54 (98%) and in only 1 case, the yeast was isolated from urine (no yeast isolated from the gut in this patient, 1/54 [6]. Among 55 patients with at least 1 ethanol producing prokaryote isolated from gut or urine, the most frequent microbes were *Candida* (80%), *Saccharomyces* (24%). The recently defined *Pichia* genus and *P. kudriavzevii* species (formerly named *Candida kruzei*) was found in only 3 cases. *Klebsiella pneumoniae* was isolated in only 2 cases with only one without any concomitant yeast [1]. Therefore, *Candida* and *Saccharomyces* yeast are much more frequent than bacteria as putative culprit of ABS. Among 49 patients for whom the fungal or bacterial species was available, *C. albicans* (n = 26, 53%) and *S. cerevisiae* (n = 9, 18%) were the 2 most frequent species identified (Supplementary Table S2).

Treatment and outcome

Treatment was available for 28 patients. In 26 cases antifungal agents were used including mainly fluconazole alone (n = 7), association of antifungal treatments (7), trichomycin (only in Japan, 6), nystatin (2), micafungin (1) and voriconazole (1). In 10 cases, low carbohydrate diet was used. Among 12 cases with detailed outcome, we found that most of them were cured with antifungal treatment and low carbohydrate diet (fluconazole (n = 4), nystatin 1, trichomycin 1 micafungin 1). One case refractory to antifungal treatment was cured with antibiotics after *K. pneumoniae* was idfentifed whereas no yeast was detected by culture nor ITS sequencing [1]. However, only 1 case was cured with this diet without antifungal treatment. Strikingly, Cordell et al. [4] reported a case with *Enterobius vermicularis* and an unidentified yeast refractory to fluconazole and nystatin pointing out the importance of microbial identification and antimicrobial sensitivity assessment. And in one case, oral fluconazole 3 weeks, nystatin 3 weeks and amphotericin B 4 weeks were ineffective after the identification of *Candida glabrata*. However, antifungal sensitivity assessment was not provided and this is important because *C. glabrata* is naturally resistant to fluconazole. To our knowledge, this is the only case in whom fecal microbiota transplantation was used and allowed complete cure of a refractory case [9].

Supplementary Table S1. Details and references of 59 cases of auto-brewery syndrome found in 55 articles from the literature

See Excel file.

Supplementary Table S2. Species associated with auto-brewery syndrome

**Species**	**N = 49**
Candida albicans	26 (53%)
Saccharomyces cerevisiae	9 (18%)
Candida glabrata	7 (14%)
Candida tropicalis	6 (12%)
Pichia kudriavzevii	3 (6%)
Candida parapsilosis	2 (4%)
Candida stelloidea	2 (4%)
Saccharomyces boulardii	2 (4%)
Candida guillIermondii	1 (2%)
Candida kefyr	1 (2%)
Candida intermedia	1 (2%)
Klebsiella pneumoniae	1 (2%)

In this table, only 49 cases in which the causative pathogen was identified to the species level were reported. The sum is greater than 49 because more than one species may be present in a single individual (see Supplementary Table S1).

Supplementary Table S3. Fecal ethanol measurements

See Excel file.

Supplementary Table S4. Results of fungal and Gram-negative bacterial culture

See Excel file.

Supplementary Table S5. Ethanol production of yeasts

See Excel file.

Supplementary Table S6. Ethanol production of Gram-negative bacterial strains

See Excel file.

Supplementary Table S7. Triglyceride production by Yeast strains from NASH patients

See Excel file.

Supplementary Figure S1. Repartition of species in 53 cases of auto-brewery syndrome in which at least 1 yeast and / or 1 bacterium was identified

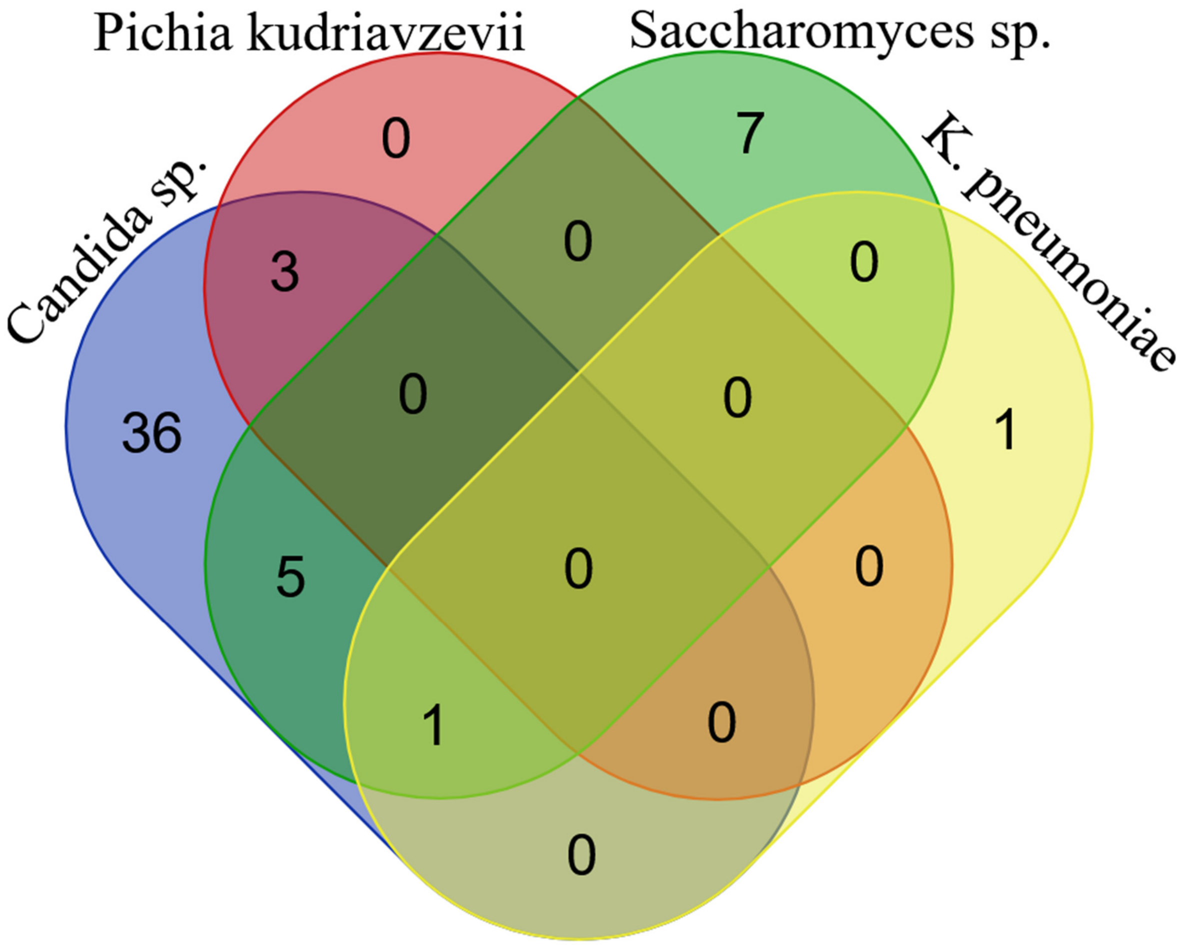

In this Venn diagram, we plotted the co-occurrences of *Candida* species, *Pichia kudriavzevii*, *Saccharomyces* yeast, and *K. pneumoniae* among 53 cases with at least 1 yeast and/or 1 bacterium identified. Intriguingly, Cordell [4] reported 1 case in which only the parasite *Enterobius vermicularis* was identified. This case was not reported in this figure.

Supplementary Figure S2. Comparison of ethanol production between NASH and controls for each species.

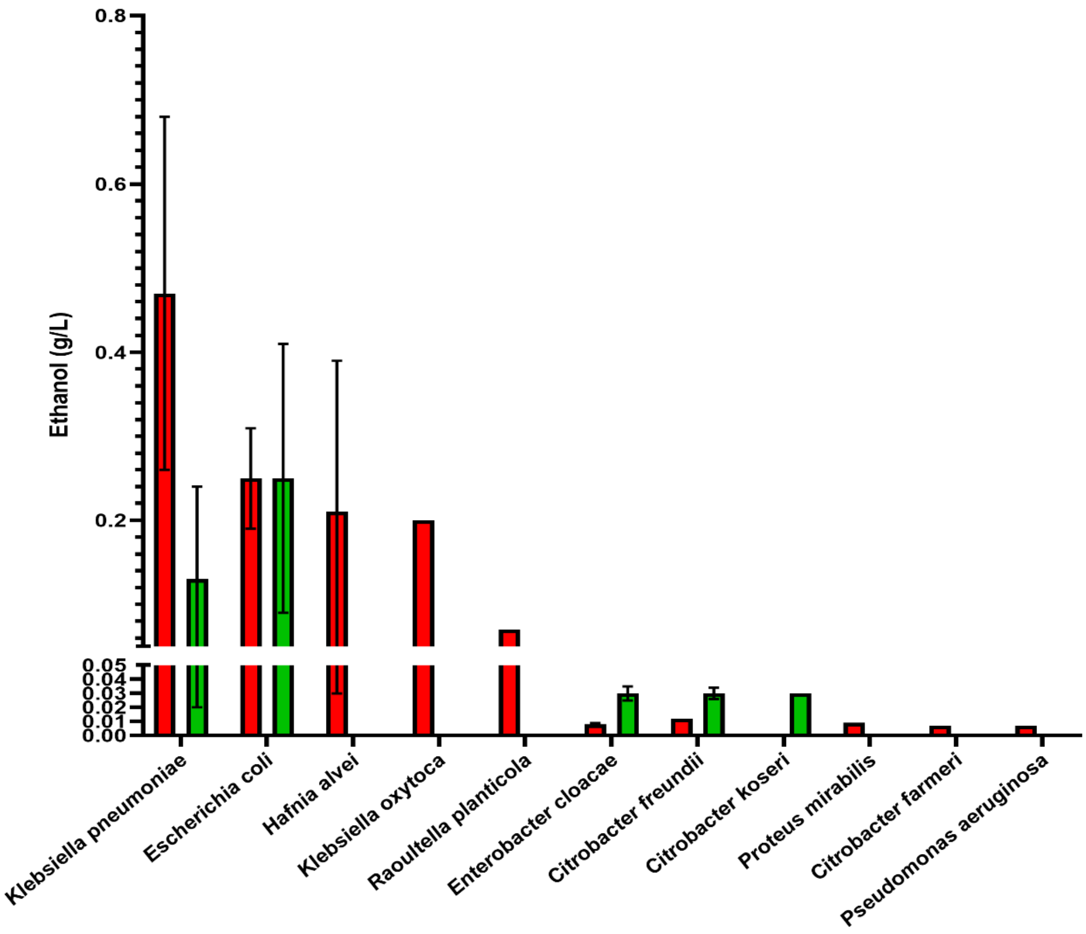

No significant differences were found.

References

1. Yuan J, Chen C, Cui J, Lu J, Yan C, Wei X, et al. Fatty liver disease caused by high-alcohol-producing Klebsiella pneumoniae [published correction appears in Cell Metab.2019 Dec 3;30:1172]. Cell Metab. 2019;30:675-88.e7.

2. Kaji H, Asanuma Y, Yahara O, et al. Intragastrointestinal alcohol fermentation syndrome: report of two cases and review of the literature. J Forensic Sci Soc. 1984;24:461-71.

3. Akbaba M. A medicolegal approach to the very rare Auto-Brewery (endogenous alcohol fermentation) syndrome. *Traffic Inj Prev*. 2020;21:295-297.

4. Cordell B, Kanodia A. Auto-Brewery as an Emerging Syndrome: Three Representative Case Studies. J Clin Med Case Reports. 2015;2: 5

5. Takahashi G, Hoshikawa K, Kan S, Akimaru R, Kodama Y, Sato T *et al.*Auto-brewery syndrome caused by oral fungi and periodontal disease bacteria. Acute Med Surg. 2021;8: e652.

6. Kruckenberg KM, DiMartini AF, Rymer JA, Pasculle AW, Tamama K. Urinary Auto-brewery Syndrome: A Case Report. Ann Intern Med. 2020;172(10):702-704.

7. Spinucci G, Guidetti M, Lanzoni E, Pironi L. Endogenous ethanol production in a patient with chronic intestinal pseudo-obstruction and small intestinal bacterial overgrowth. Eur J Gastroenterol Hepatol. 2006;18:799-802.

8. Guo X, Zhang W, Huang R, Ma J, Liu Z, Hu D, et al. The case study of one patient with gut fermentation syndrome: case report and review of the literature. Int J Clin Exp Med. 2018;11:4324-29.

9. Vandekerckhove E, Janssens F, Tate D, De Looze D. Treatment of gut fermentation syndrome with fecal microbiota transplantation. Ann Intern Med. 2020;173:855.
